# Artemisinin Combination Therapy: A Good Antimalarial, but Is the Dose Right?

**DOI:** 10.1371/journal.pmed.1001565

**Published:** 2013-12-03

**Authors:** Paul Garner

**Affiliations:** Liverpool School of Tropical Medicine, Liverpool, United Kingdom

## Abstract

In an accompanying Perspective, Paul Garner further discusses dosing issues for artemisinin combination therapy.

*Please see later in the article for the Editors' Summary*

**Linked Research Article**

This Perspective discusses the following new study published in *PLOS Medicine*:

WorldWide Antimalarial Resistance Network (WWARN) DP Study Group (2013) The Effect of Dosing Regimens on the Antimalarial Efficacy of Dihydroartemisinin-Piperaquine: A Pooled Analysis of Individual Patient Data. PLoS Med 10(12): e1001564. doi:10.1371/journal.pmed.1001564

Ric Price and colleagues pool individual patient data from efficacy trials of dihydroartemisinin-piperaquine shared with WWARN (Worldwide Antimalarial Resistance Network) to examine the potential for underdosing in young children.

Artemisinin combination therapy (ACT) has revolutionised malaria treatment. ACTs combine an artemisinin derivative (a relatively new group of very effective drugs [1]) with another longer-lasting drug from another class to try to reduce the risk of further resistance developing. ACTs cure over 90% of people; they also act against malaria gametocytes, so potentially reduce transmission [1].

In 2006, the World Health Organization (WHO) recommended ACTs for uncomplicated *Plasmodium falciparum* malaria worldwide [2]. In 2010, the WHO added dihydroartemisinin-piperaquine (DHA-PPQ) to their existing list of four recommended ACTs (that is, artemether-lumefantrine, artesunate-amodiaquine, artesunate-mefloquine, and artesunate-sulfadoxine-pyrimethamine). DHA-PPQ is clearly at least as good as existing options, and it has a simple dosing schedule of one dose daily for three days [3],[4].

## Dosing Bands in Children

One problem with DHA-PPQ is that the drug ratio between the two components is not ideal, because the level of DHA in the fixed-dose product that has received regulatory approval is probably too low [5]. In addition, the approved weight-based schedules mean that some children may not receive the WHO-specified minimum daily doses of either drug, depending on whether a child's weight is close to the upper cut-off for a particular dose category. Thus, some children do not receive the minimum daily dose set for PPQ, which has a narrower therapeutic index, whereas some children receive a dose very close to the minimum daily dose for DHA. Indeed, a higher DHA∶PPQ ratio in the formulation would have reduced the under-dosing with DHA, which has a much wider therapeutic index anyway.

These dosing problems related to the ratio of components and the weight cut-offs for whole and half tablet dosing are not the only challenges with getting children the appropriate antimalarial treatment. Whilst clinical trials carefully weigh children and dose them accordingly, most routine health clinics use age to determine the dose. Since the relationship between weight and age varies among children and localities, this increases the chances of under-dosing [6]. Children are growing rapidly at this time, so age is rounded down, and this risk is further increased. This problem is further complicated by questions raised about dose in a pharmacokinetic study in young children from Burkina Faso, suggesting that PPQ levels in younger children reach lower concentrations for a given dose per kilogram, indicating that children may need a higher PPQ dose relative to adults [7]. Specialists are concerned because earlier work has shown that lower plasma levels of PPQ are clearly associated with increased risk of failure [8].

## Dose and Failed Treatment

An individual patient data-level meta-analysis by Ric Price and colleagues [9] in this week's *PLOS Medicine* provides substantially new information about the extent of the dosing problem and its consequences. The authors pooled data from 26 trials across more than 7,000 participants and were able, using data from trial arms receiving DHA-PPQ, to estimate that the percentage of children whose total PPQ dose fell below WHO recommended lower limits was about 29%. This high rate of under-dosing occurred even under the ideal conditions of carefully managed controlled trials using predominantly weight-based schedules. After adjusting for confounders, the mg/kg dose of PPQ was shown to be a significant predictor for recrudescence. Although the actual absolute cure rates are still above 90%, treating malaria on a wide scale with suboptimal doses of antimalarial drugs with associated treatment failure is likely to contribute to selection and spread of drug-resistant parasites.

## Methods Innovation

The analysis by Price and colleagues is impressive and appropriate to the question being tackled. The word “meta-analysis” and randomised controlled trials usually implies relative differences between randomised groups, but this study is different. Here the authors have brought together the individual data from comparative and non-comparative trials, and analysed the outcomes—mostly measures of cure—in relation to risk factors, such as age, malaria endemicity, and dose of drug per kilogram of body weight. The authors ignore the randomisation and analyse the data as an observational dataset. This is different to conventional analysis of the randomised comparisons. In the work of the Cochrane Infectious Diseases Group in malaria (which I co-ordinate), we have been preparing and updating reviews of malaria since the mid -1990s. We are able to give robust messages that guide policy, but we observe substantive quantitative heterogeneity between trials that is not explained by subgroup analysis. This does not impair the conclusions about comparative effects, but leaves us wondering how to get to grips with all the factors—dose, host immunity, parasite drug resistance—that might influence the absolute cure rate. By analysing trial arms observationally, Price and colleagues can examine patient factors and directly relate this to individual outcomes. This is innovative and useful, drawing on the rigor of the trial design in standardising data collection, and is complementary to existing efforts.

## Substantial Effort

This individual patient data analysis represents an early and important output of the Worldwide Antimalarial Resistance Network (WWARN). This global network was initially set up to monitor drug resistance, but the opportunities to explore risk factors for treatment failure, such as dose (as in Price et al.), are now becoming obvious. To do this, the investigators have had to set up formal data-sharing legal agreements with all the investigators carrying out malaria trials, and to agree on standard outcomes, measurements, and procedures. It is not surprising this network has taken some years to establish, and the extensive, formal collaboration between researchers is impressive.

Creation of the network has been helped by the establishment, some years ago, of fairly standard efficacy outcomes across malaria trials. However, adverse event detection and reporting has generally not been standardised between trials; it is often done badly and to date has not been part of WWARN's brief. Likely as a result, while Price and colleagues report on gastrointestinal toxicity, it is not clear if the data were simply tolerability data collected in efficacy trials to make sure everyone got an adequate dose or formal adverse event data collection, and over half the trials do not contribute to this analysis. Nevertheless, this is less a problem with WWARN and more a generic problem due to the lack of adverse event standardisation between trials in malaria—and probably more generally. However, if the Network and analyses of this type are going to examine dose optimisation, then standardising the collection and reporting of and rare adverse events, including cardiotoxicity, will be important.

## What's Next?

There is no doubt that some dose optimisation is required, and dosing schedules will need to be changed so that children at the lower end of the dosing band per kilogram receive optimal doses. This dosing adjustment will require careful collection of toxicity and adverse event data, and one trial is currently recruiting to do this [10]. Furthermore, optimising dosing does not take into account the translation from weight- to age-based dosing bands for programmatic implementation.

An additional consideration is that some advocates are promoting programmes that treat everyone in a population for malaria (or test everyone and treat those positive) to attempt to eradicate malaria [11]. If DHA-PPQ is an option for these policies, then getting the dose right is particularly important. There is a balancing act between under-dosing, which increases the risk of resistance developing, and increasing dosing, such that toxicity and adverse events increase. This trade-off is particularly important for mass treatment of whole populations because the drug is being used to treat children who may not even be infected, so the benefit–risk balance is different than when treating sick children.

More broadly, this is not the first time that under-dosing in children has been shown to be a problem associated with fixed-dose combinations: in 2006, WHO revised its dosing for ethambutol for the treatment of tuberculosis, which led to a change in recommendations for the fixed-dose combination product [12]. Researchers need to focus on age-based dosing and the practical problems with banding. Better attention to dosing and formulation early in the drug development cycle, as well as considering how this then translates to age-based dosing schedules, are important to ensure that children are cured, adverse effects are avoided, and drug resistance is prevented as much as possible. After all, this was the starting point for developing ACTs.

## Abbreviations

ACT: artemisinin combination therapy
DHA: dihydroartemisinin
PPQ: piperaquine
WHO: World Health Organization
WWARN: Worldwide Antimalarial Resistance Network
